# Identification of three molecular subtypes based on immune infiltration in ovarian cancer and its prognostic value

**DOI:** 10.1042/BSR20201431

**Published:** 2020-10-28

**Authors:** Juan Liu, Zongjian Tan, Jun He, Tingting Jin, Yuanyuan Han, Li Hu, Jukun Song, Shengwen Huang

**Affiliations:** 1 School of Medicine, Guizhou University, Guiyang, China; 2 Department of Reproductive Medicine, Guizhou Provincial People’s Hospital, Guiyang, China; 3 Prenatal Diagnosis Center, Guizhou Provincial People’s Hospital, Guiyang, China; 4 Department of Oral and Maxillofacial Surgery, Guizhou Provincial People’s Hospital, Guiyang, China; 5 NHC Key Laboratory of Pulmonary Immunological Diseases, Guizhou Provincial People’s Hospital, Guiyang, China

**Keywords:** CIBERSORT algorithm, Immunotherapy, Ovarian cancer, Prognostic value, Tumor-infiltrating immune cells

## Abstract

Background: Increasing studies suggest that tumor immune infiltration is a relative factor of prognosis in ovarian cancer (OvCa). The present study explored the composition of tumor-infiltrating immune cells (TIICs) in OvCa using CIBERSORT algorithm and further assessed their values for prognosis and therapeutic strategies by molecular subtypes.

Methods: Publicly available databases including The Cancer Genome Atlas (TCGA) and GTEx were searched. Ovarian tumor samples were available from TCGA, and normal ovarian samples were obtained from the GTEx dataset. The relative proportions of immune cell profiling in OvCa and normal samples were evaluated by CIBERSORT algorithm. Association between each immune cell subtype and survival was inferred by the fractions of 22 immune cell types. “CancerSubtypes” R-package was employed to identify the three types of molecular classification and analyze the functional enrichment in each subclass. Response to immunotherapy and anticancer drug targets was predicted via TIDE algorithm and GDSC dataset.

Results**:** Substantial variation reflecting individual difference was identified between cancer and normal tissues in the immune infiltration profiles. T cells CD4 memory activated, macrophages M1 were associated with improved overall survival (OS) as evaluated by univariate Cox regression and multivariate Cox. Three subtypes were identified by ´CancerSubtypes’ R-package and every sub-cluster possessed specific immune cell characterization. Meanwhile, Cluster II exhibited poor prognosis and sensitive response to immunotherapy.

Conclusions**:** The cellular component of immune infiltration shows remarkable variation in OvCa. Profiling of immune infiltration is useful in prediction of prognosis of OvCa. The results from profiling might be considered in therapeutic modulation.

## Introduction

Ovarian cancer (OvCa) is the fifth leading cause of cancer-related deaths in women with 295,414 new cases and 184,799 estimated deaths in 2018 [1,2]. Given that the ovaries are located in the pelvic cavity, the early symptoms are not evident, and approximately half of patients are diagnosed in the late stage. Therefore, OvCa has the worst prognosis among all gynecological tumors. In most countries, the 5-year survival rate is still less than 45% [3]. Despite remarkable advances in surgery and chemotherapy, considerable interspace still exists for prognosis improvement. Studies have found that ovarian cancer is an immunogenic disease, so recognizing the roles of immune cells in the tumor-related microenvironment during tumor development is very important [4,5].

Tumor-infiltrating immune cells (TIICs), which include B cells, T cells, dendritic cells (DCs), macrophages, neutrophils, monocyte and mast cells, can regulate cancer progression and facilitate potential therapeutic targets [6]. Numerous studies have reported the important roles of TIICs on cancer development [7–9]. However, the specific mechanism involved in OvCa development is still unclear. The CIBERSORT method is a gene-based deconvolution algorithm that can assess the immune cell compositions of complex tissues on the basis of gene expression profiles from bulk tumor samples [10]. In the present study, CIBERSORT was employed to define the 22 TIIC subtypes of the immune response in OvCa and uncover the interaction effect with molecular subtypes and clinical features. Our results also indicate that the molecular subclasses of OvCa possess distinct immune phenotypes. The current research also provides theoretical foundation for immunotherapy strategy against OvCa.

## Materials and methods

### Data source and processing

RNA-seq data (FPKM values) of 379 high-grade serous OvCa samples were downloaded from the Cancer Genome Atlas (TCGA) website (https://cancergenome.nih.gov/). mRNA profiles (FPKM values) of 88 normal ovarian tissues were obtained from the GTEx project (https://commonfund.nih.gov/GTEx/). These two types of mRNA data were merged into one gene expression profile using comBat algorithm in the ´sva’ of R package to eliminate the batch effects. Voom standardized method (variance modeling at the observational level) was employed to normalize the RNA-seq profiles.

### Quantification of TIICs using the CIBERSOFT algorithm

The CIBERSOFT method using gene expression data was applied to deduce 22 human immune cell types and manipulate the features of 547 marker genes to quantify the relative scores for each cell type. The deconvoluted *P* value for each sample was derived using Monte Carlo sampling through CIBERSORT algorithm to improve result accuracy. The standardized processed dataset of gene expression was uploaded to the CIBERSORT website (https://cibersort.stanford.edu/index.php) which running with 1000 aligned default signature matrices.

### Survival analysis

Univariate Cox and Kaplan–Meier survival analyses were employed to screen the prognostic 22 human immune cell phenotypes by the ´survival’ package in R Software for leukocyte signature matrix (LM22) immune cells and OS. Multivariate Cox regression analysis was selected to further identify the prognostic 22 human immune cell phenotypes as prognosis factors. In addition, the correlation between LM22 immune cell and grade stage was analyzed.

### Identification of molecular subtypes

Consensus cluster algorithm was analyzed using ´CancerSubtypes’ R-package [11] to calculate the number of clusters in the tumor samples and further explore different tumor microenvironment (TME) cell infiltration patterns. Differentially expressed genes (DEGs) were identified using the Limma package of R software to reveal the latent difference in TME cell infiltration models. Data sets with |log2 fold change| ≥ 0.1 and *P* value less than 0.05 for subsequent analysis were selected as criteria.

### Analysis of different functional and pathway enrichment in OvCa subtypes

´ClusterProfiler’ R package was used to reveal the potential biological significance of DEGs among TME subtypes, Gene Ontology (GO) biological process terms, and Kyoto Encyclopedia of Genes and Genomes (KEGG) pathway [12]. *P* value <0.05 and false discovery rate (FDR) of <0.05 were the threshold for GO enrichment analysis. Gene Set Enrichment Analysis (GSEA) was applied to reveal the integral pathway of gene set activity score for each sample [13]. The gene sets were downloaded using the c2 curated signatures from the Molecular Signature Database (MSigDB) of Broad Institute. Data sets with |logFoldChange| ≥0.2 and adjust *P* value <0.05 were considered as the significant enrichment pathway.

### Prediction for response to immunotherapy or anti-cancer drug in OvCa subtypes

Immunotherapeutic response was predicted by tumor immune dysfunction and exclusion (TIDE) algorithms [14] and subclass mapping [15]. The pharmacy medicine response of subtype samples was also predicted using the public pharmacogenomics database [Pharmaceutical Sensitivity Genomics in Cancer (GDSC) https://www.cancerrxgene.org/] [16]. pRRophetic was employed for the prediction process. Half-maximal inhibitory concentration (IC50) and prediction accuracy were estimated by ridge regression and 10-fold cross-validation relying on the GDSC training set, respectively [17].

### Statistical analysis

The main study included the patients with a CIBERSOFT *P* value less than 0.05. After initial screening, the samples were divided into two groups: OvCa tumor samples and normal samples. The outline of immune cells was assessed for each sample. Pearson correlation analysis was conducted to discover the interrelation among the 21 immune cell types between the two groups. Statistical significance was detected using Wilcox test for comparisons between two groups and Kruskal–Wallis tests for more than two groups [18]. Relationship between the proportions of immune cell type and survival was examined through Cox regression analysis. The differences in OS among groups were tested through log-rank statistic using Kaplan–Meier plots [19]. Variables with a *P*<0.05 were regarded as independent prognostic OS factors in the univariate Cox regression analysis. The included prognostic factors were applied to construct the multivariate Cox regression model for OS and the variables were adjusted at age, FIGO stage and tumor grade. Wilcox test was used to assess the association between various immune cell subtypes. R-Language (R-project.org) and packages obtained through the Bioconductor project (www.bioconductor.org) were applied for statistical analysis. Clinicopathologic characteristics were compared among the three subtypes using the χ^2^ test, and Fisher’s exact test was added when necessary by SPSS version 22.0 statistical software. All *P* values were set as bilateral, and a *P* value <0.05 was regarded as statistically significant.

## Results

### Overview of data

Among the 467 samples, 88 normal patient samples were obtained from GTEx dataset and 379 tumor samples were obtained from TCGA dataset. After CIBERSOFT algorithm was applied, 451 patients (26 normal patients and 325 tumor patients) with a *P* value <0.05 were considered as eligible.

### Profile of TME in OvCa and clinicopathologic characteristics of TME subtypes

**Figure 1:**
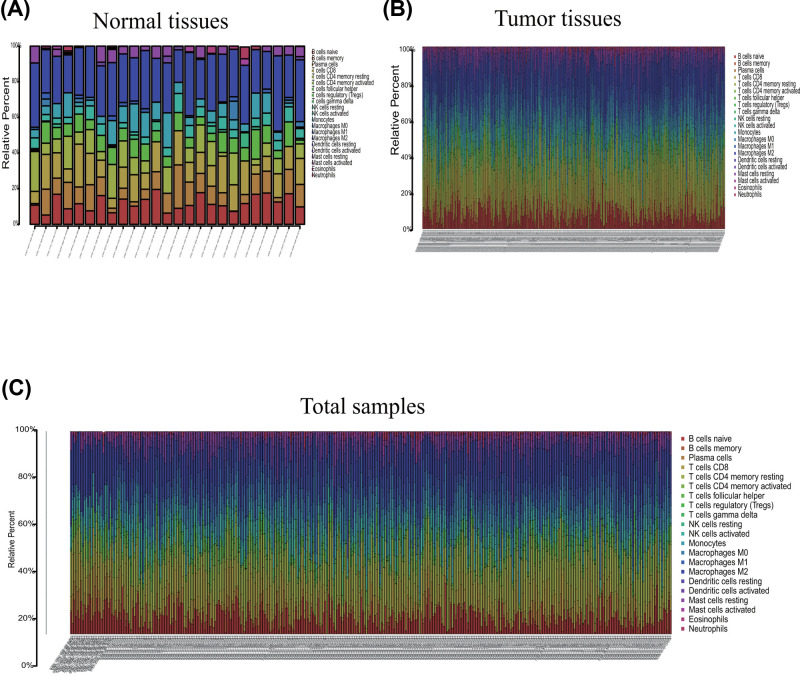
Composition of TIICs in OvCa tissues and normal tissues with CIBERSOST (**A**) Relative proportions of 22 TIIC subpopulation in normal samples. (**B**) Relative proportions of 22 TIIC subpopulation in OvCa samples. (**C**) Relative proportions of 22 TIIC subpopulation in total samples.

**Figure 2:**
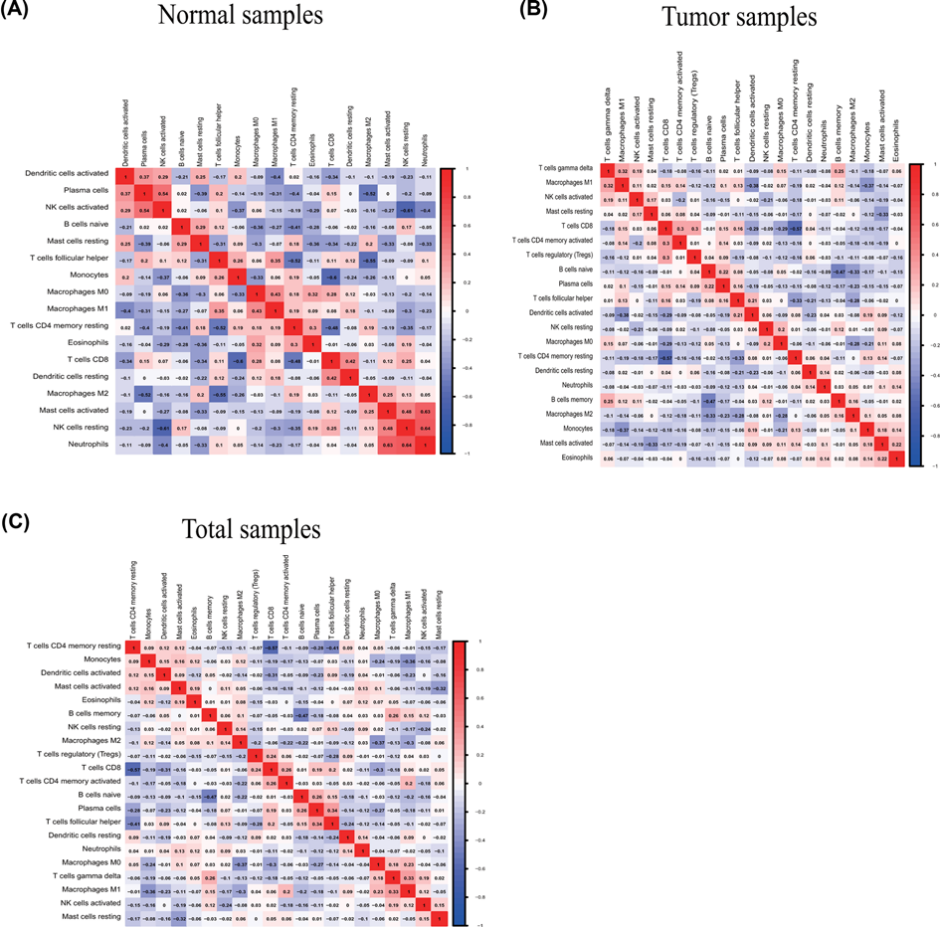
Correlation matrix of all immune proportions and Immune cell interactions in the TCGA ovarian cancer cohort Panels (**A-C**) are for normal, tumor, and total samples, respectively.

**Figure 3:**
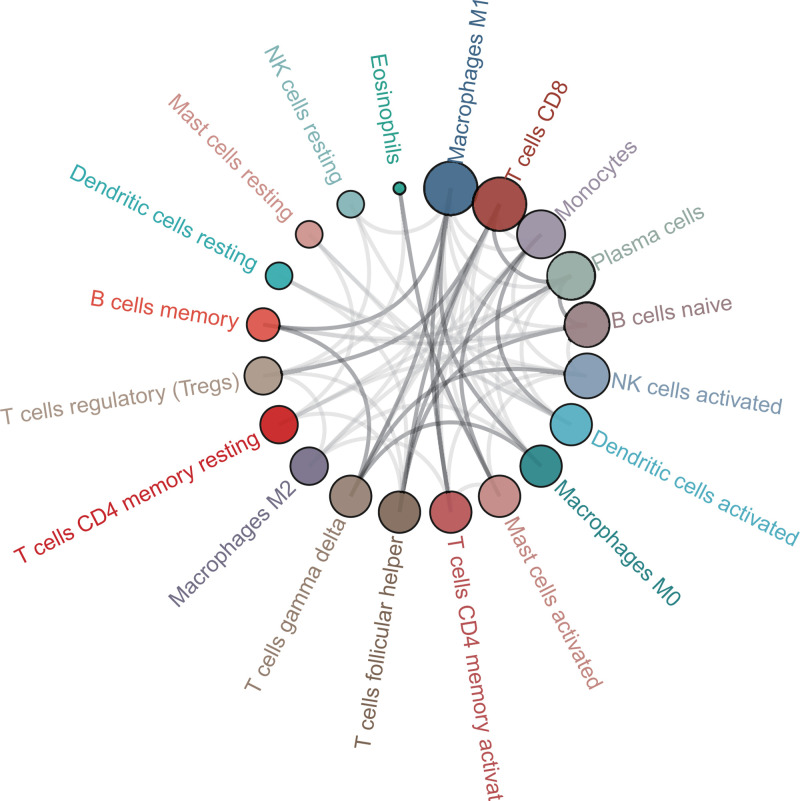
Correlation analysis of immune cells in tumor samples

**Figure 4:**
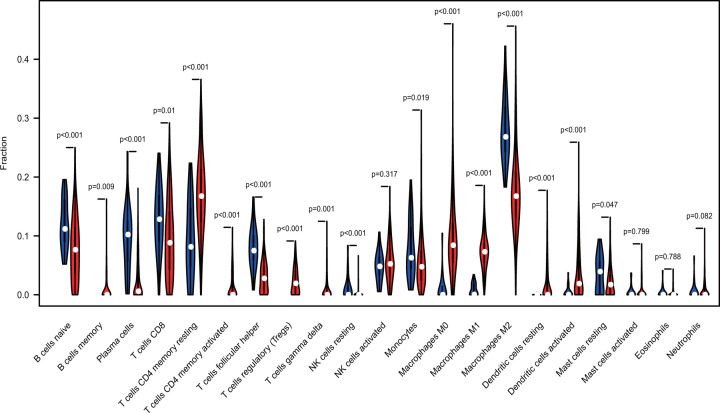
Violin plot indicating the difference of CIBERSOFT immune cell fractions between tumor and normal tissues

The profile of TME cell infiltration models and TME signatures was systematically evaluated by CIBERSOFT algorithm. The fraction of T cells CD4 naÏve in all samples was zero after data filtration, so T cells CD4 naÏve was excluded in the subsequent analysis and 21 immune cell types were taken into account. Figure 1 summarizes the findings from the 26 normal samples and 325 tumor samples. Figure 2A–C showed several strongly positive correlation pairs among the 21 types of immune cells in the normal samples. However, the mutual relationship among LM21 immune cells was weakened in the tumor samples. For example, NK cells resting was highly positive related with Neutropils in the TMC from the normal samples, and NK cell resting was highly negatively associated with NK cells activated in the normal group. T cells CD8 were strongly negatively related to T cells CD4 memory resting in the ovarian tumor group. Therefore, the variations in the TME cell infiltration proportion can reflect the difference in immunity between the two groups. The immune cell correlation in the tumor samples indicated that T cells CD8 and Macrophages M1 are strongly correlated with other immune cells (Figure 3). As shown in Figure 4, we found that the fraction of most of the LM22 immune cells was different between normal and tumor groups. Such as B cells naive, B cells memory, plasma cells, T cells CD8, T cells CD4 memory resting, T cells CD4 memory activated, T cells follicular helper, T cells regulatory (Tregs), T cells gamma delta, NK cells resting, Monocytes, Macrophages M0, Macrophages M1, Macrophages M2, Dendritic cells activated, Dendritic cells resting and Mast cells resting were markedly different between normal and tumor group, Just NK cells activated, Mast cells activated, Eosinophils and Neutrophils were not obviously changed between two groups.

### Correlation with other clinical factors

**Figure 5:**
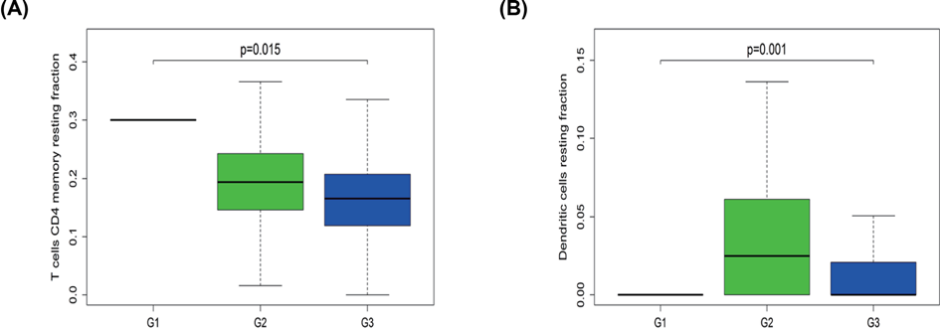
Association between clinical feature and immune cells (**A**) Fraction of T cells CD4 memory resting was higher in G1/2 than in G3 tumors. (**B**) Dendritic cells resting were fewer in G3 than in G2 tumors.

In terms of pathological stage, the fraction of T cells CD4 memory resting and Dendritic cells resting was higher in the early stage of OvCa (G1/G2) than in the late stage (G3) (Figure 5A,B). These results revealed that aberrant immune infiltration might play important roles in tumor development and therefore has clinical importance.

### Screen of prognostic LM22 immune cell subtypes

**Figure 6:**
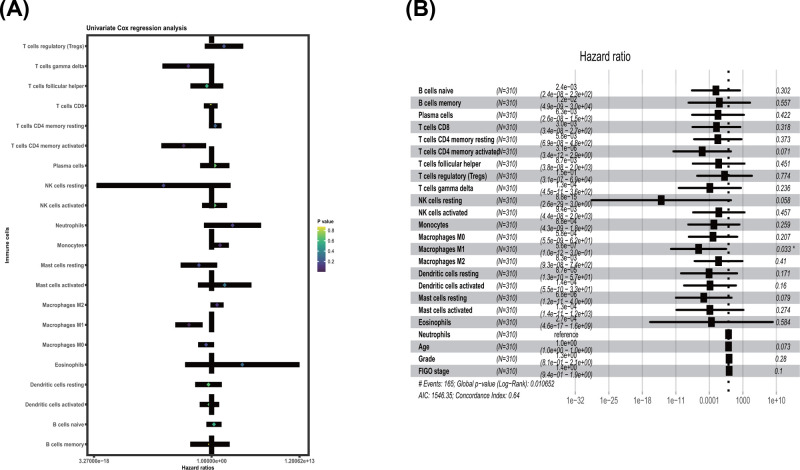
Prognostic association between survival and immune cells in TCGA ovarian cancer cohort was conducted (**A**) Univariate Cox regression analysis; (**B**) Multivariate Cox regression analysis.

Univariate Cox regression was applied to test the prognostic LM21 immune cell subtypes in all tumor samples. T cells CD4 memory activated, Macrophages M1 were significantly correlated with improved OS (*P* value <0.05) (Table 1 and Figure 6A). Multivariate Cox proportional hazard regression analysis was also performed to further distinguish the prognostic LM21 immune cell subsets. Followed with adjusting at age, FIGO stage and tumor grade in multivariate analyses, the results showed that the Macrophages M1 were associated with improved outcome, and this finding is similar to that of univariate Cox regression (Table 2, Figure 6B).

**Table 1:** The prognostic value of TIICs subtypes was assessed via univariate Cox regression analysis in OvCa

Immune cell	HR	HR.95L	HR.95H	*P* value
B cells naive	2.259857742	0.167965175	30.40485632	0.538709079
B cells memory	0.570289666	0.000665353	488.8086572	0.87052972
Plasma cells	2.97136951	0.019594531	450.5867841	0.670793633
T cells CD8	0.750490394	0.071509509	7.876376675	0.810874328
T cells CD4 memory resting	3.471684378	0.405593057	29.71597322	0.25587735
T cells CD4 memory activated	7.11E-05	3.27E-08	0.15468352	0.014852893
T cells follicular helper	0.196952671	0.000706208	54.92766094	0.571696164
T cells regulatory (Tregs)	69.54558716	0.096554183	50091.9643	0.206367735
T cells gamma delta	0.000348359	4.77E-08	2.541556599	0.07935633
NK cells resting	7.12E-08	7.49E-18	677.2261025	0.160340392
NK cells activated	2.747675411	0.038022159	198.5610567	0.643490667
Monocytes	18.35771465	0.900479984	374.2511697	0.058515501
Macrophages M0	0.152919343	0.00990954	2.35977909	0.178621356
Macrophages M1	0.000429741	3.93E-06	0.046985602	0.001209318
Macrophages M2	6.486385634	0.679602626	61.90852856	0.104291777
Dendritic cells resting	0.341577328	0.003582433	32.56866906	0.644116459
Dendritic cells activated	0.474828542	0.017029143	13.23978209	0.660927178
Mast cells resting	0.013068359	2.51E-05	6.814648616	0.174212647
Mast cells activated	88.7341843	0.009262197	850095.9048	0.33755272
Eosinophils	36801.78831	0.000112806	1.20062E+13	0.293193249
Neutrophils	1306.080287	0.073671392	23154791.34	0.150594407

**Table 2:** The prognostic value of TIICs subtypes was estimated via multivariate Cox regression analysis in OvCa

Immune cell	HR	HR.95L	HR.95H	*P* value
B cells naive	0.002355227	2.42E-08	229.4149562	0.301837664
B cells memory	0.012119288	4.95E-09	29688.13038	0.556582545
Plasma cells	0.006277743	2.65E-08	1488.744868	0.421965348
T cells CD8	0.003018227	3.40E-08	267.8040723	0.318139601
T cells CD4 memory resting	0.005769319	6.88E-08	483.6199618	0.372778382
T cells CD4 memory activated	3.15E-06	3.37E-12	2.94370557	0.070911138
T cells follicular helper	0.008667392	3.78E-08	1985.552642	0.450823493
T cells regulatory (Tregs)	0.1473421	3.15E-07	69004.01933	0.77376096
T cells gamma delta	0.000126651	4.50E-11	356.8316428	0.236283082
NK cells resting	8.76E-15	2.57E-29	2.986266805	0.05797533
NK cells activated	0.009447908	4.39E-08	2032.559815	0.456793134
Monocytes	0.000875738	4.34E-09	176.7010177	0.258608158
Macrophages M0	0.000583426	5.48E-09	62.08253292	0.207342889
Macrophages M1	5.61E-07	1.04E-12	0.301634702	0.03251739
Macrophages M2	0.00828117	9.27E-08	739.6506108	0.409836857
Dendritic cells resting	8.73E-05	1.33E-10	57.25065617	0.171417213
Dendritic cells activated	0.000135608	5.49E-10	33.49341891	0.159807772
Mast cells resting	6.84E-06	1.16E-11	4.020557409	0.079314062
Mast cells activated	0.000128721	1.39E-11	1190.351727	0.273695984
Eosinophils	0.000273426	4.65E-17	1609199822	0.584454031
Neutrophils	NA	NA	NA	NA
Age	1.014046357	0.998710627	1.029617576	0.0728096
Grade	1.305454446	0.805252099	2.116369909	0.27956415
FIGO stage	1.353445322	0.943831044	1.940828555	0.099837957

**Figure 7:**
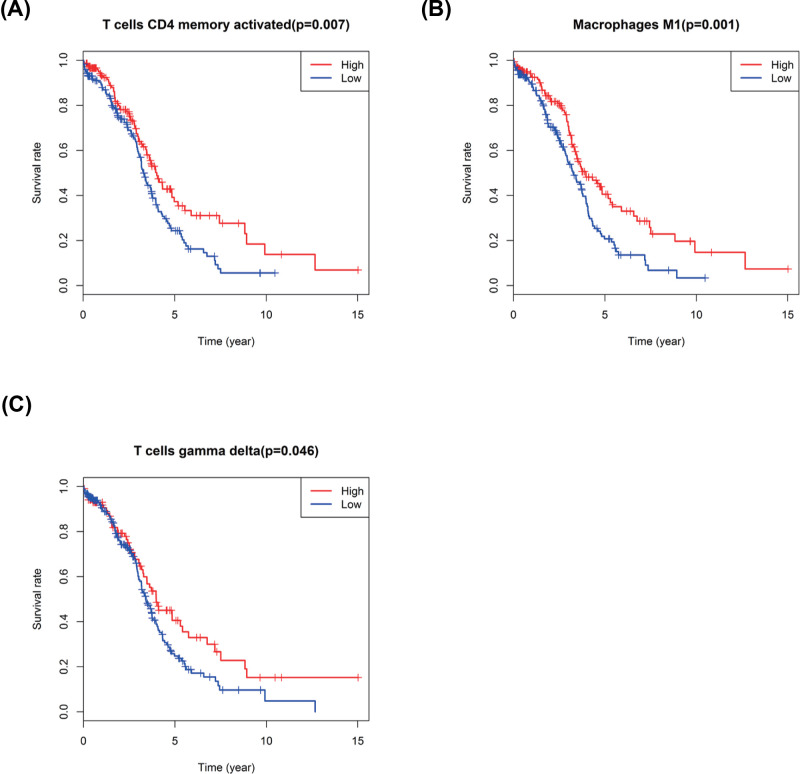
Kaplan–Meier survival analysis of three immune cells (**A**) T cells CD4 memory activated; (**B**) Macrophages M1; (**C**) T cells gamma delta.

Kaplan–Meier curve plot and log-rank test were further used to identify immune cell types, and the result showed that the T cells CD4 memory activated, T cells gamma delta and Macrophages M1 were strongly related with improved OS in patients with OvCa (Figure 7A–C).

### Identification of OvCa molecular subtypes

**Figure 8:**
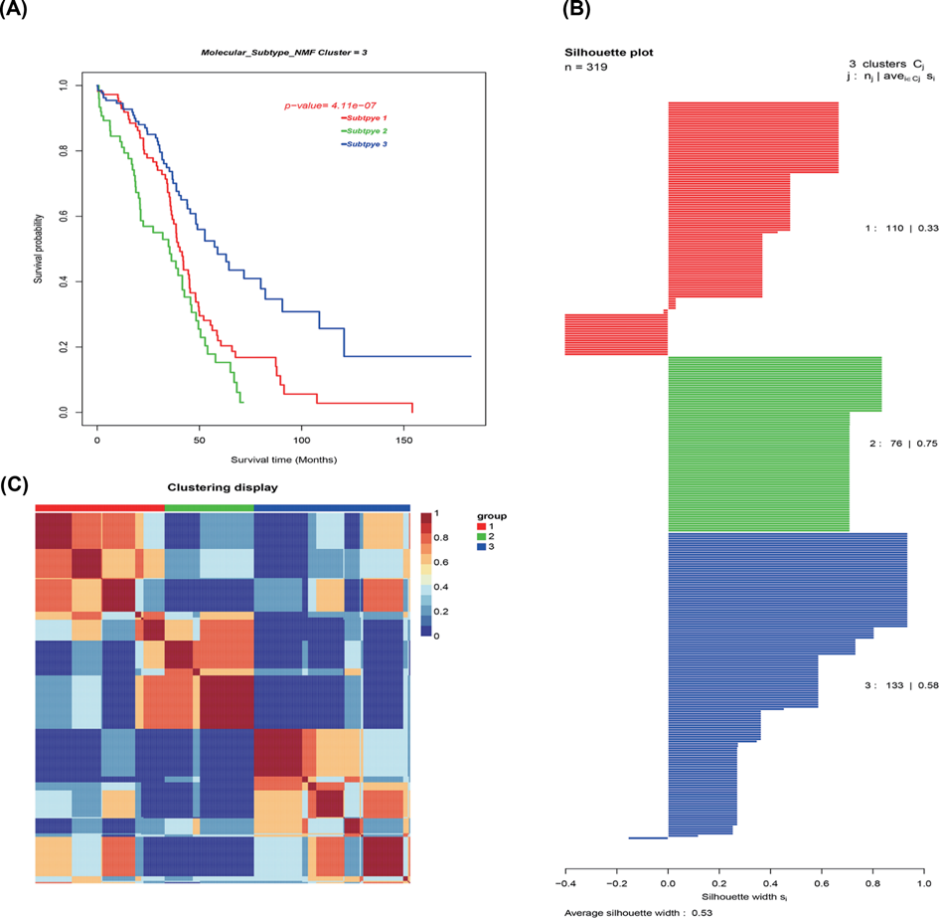
Identification of ovarian cancer molecular subtypes utilizing SNFCC+ algorithm (**A**) Log-rank test *P* value for Kaplan–Meier survival analysis. (**B**) Average silhouette width reflecting the coherence of clusters. (**C**) Clustering heatmap exhibiting the extent of the partitioning of the sample clusters.

**Figure 9:**
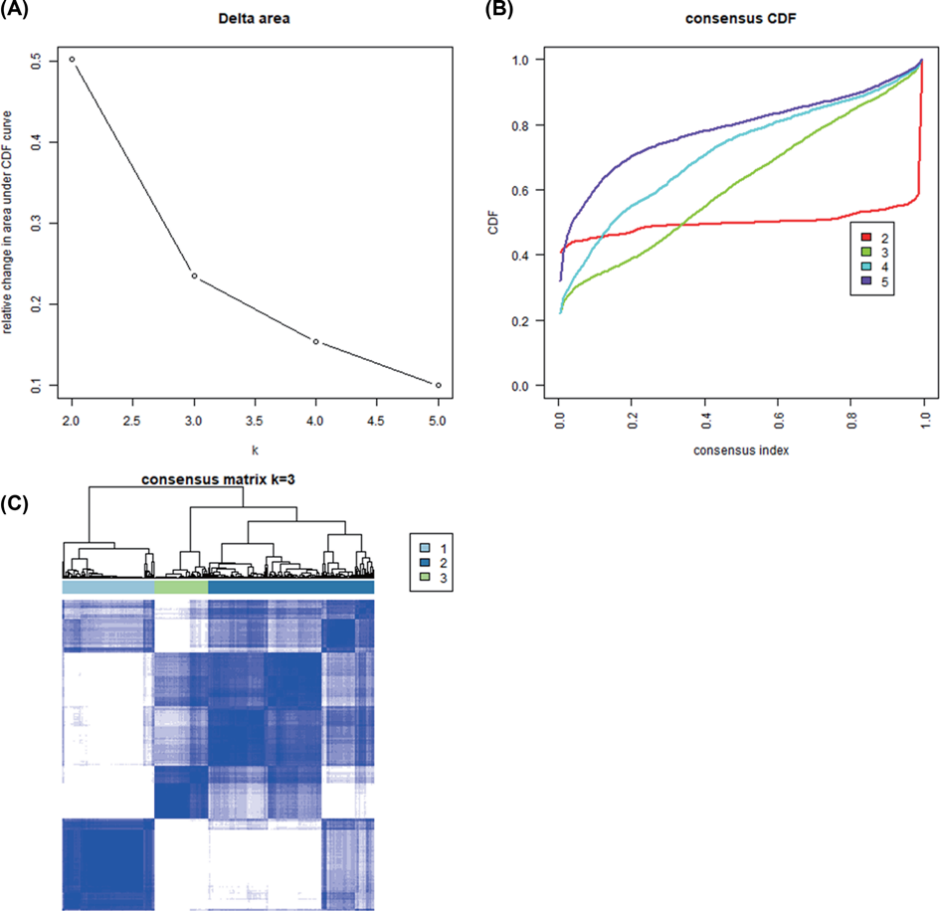
Unsupervised clustering analysis (**A**) Relative change in area under the CDF curve of *K*=2–5; (**B**) CDF curve of *K*=2–5; (**C**) Consensus heatmap

OvCa molecular subtypes were identified by utilizing unsupervised consensus clustering in all tumor samples. The optimal number of clusters was determined according to the *K* value. Three-cluster solution (*K*=3) with no remarkable augment in area under the cumulative distribution function (CDF) curve was employed, followed by the evaluation of relative variations in the area under CDF curve and the consensus heat map (Figure 8A–C). The findings classified 110 patients (34.5%) in Cluster I, 76 patients (23.8%) in Cluster II, and 133 patients (41.7%) in Cluster III for the OvCa cohort. The consensus matrix heat map clearly illustrated the Clusters I, II, and III in the individualized clusters in Figure 9A–C. Among the three clusters Cluster III exhibited the greatest prognosis, whereas Cluster II had the worst prognosis (*P*<0.001, log-rank test).

**Figure 10:**
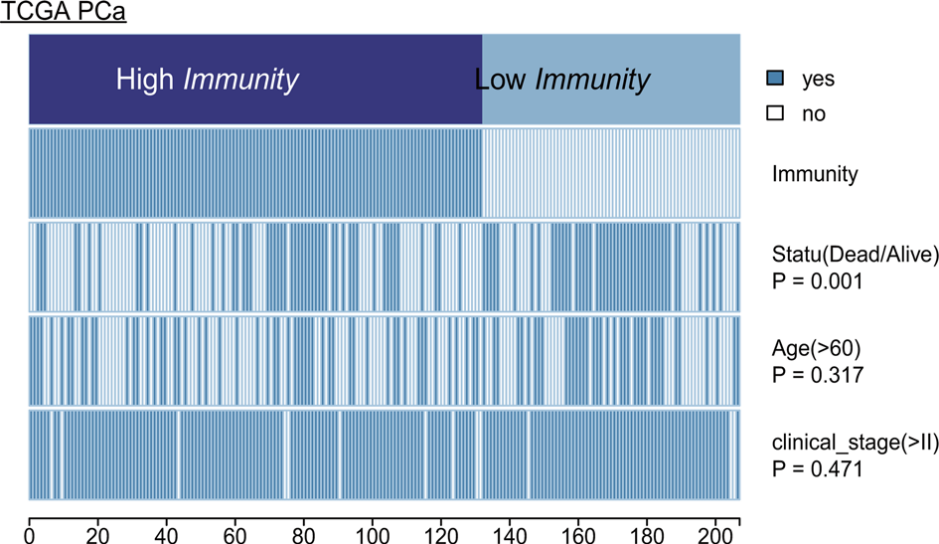
Heatmap depicting the association of disparate clinical characters with ovarian cancer subsets

In terms of clinical parameters, patient status revealed significant difference between Clusters III and II. However, the patient’s age and clinical stage of OvCa showed no significant difference. The heatmap illustrates the association of disparate clinical characters among the three subclasses (Figure 10). Statistical significance was evaluated by the Kruskal–Wallis test. Clinicopathologic characteristics were compared among the three subtypes, and the results are shown in Table 3.

**Table 3:** Comparison of clinicopathologic characteristics among three molecular subtypes in OvCa

Variables	Cluster I (*n*=110)	Cluster II (*n*=76)	Cluster III (*n*=133)	*P* value
Age at diagnosis (>60)	46(41.8%)	38(50.0%)	57(42.9%)	0.500
White (Race)	95(86.4%)	66(86.8%)	119(89.5%)	0.732
Stage II (FIGO Stage)	4(3.6%)	3(3.9%)	10(7.5%)	0.337
Stage III (FIGO Stage)	83(75.5%)	56(73.7%)	108(81.2%)	0.379
Stage IV (FIGO Stage)	21(19.1%)	16(21.1%)	14(10.5%)	0.075
G2 (histology grade)	13(11.8%)	7(9.2%)	16(12.0%)	0.806
G3 (histology grade)	94(85.5%)	66(86.8%)	115(86.5%)	0.958
3-year survival rate	44(40.0%)	20(26.3%)	61(45.9%)	0.108
5-year survival rate	13(11.8%)	6(7.9%)	21(15.8%)	0.243
Alive (Survival state)	41(37.3%)	27(35.5%)	81(60.9%)	<0.001

### Disparity of immune cell patterns of clusters I, II, and III subtypes

**Figure 11:**
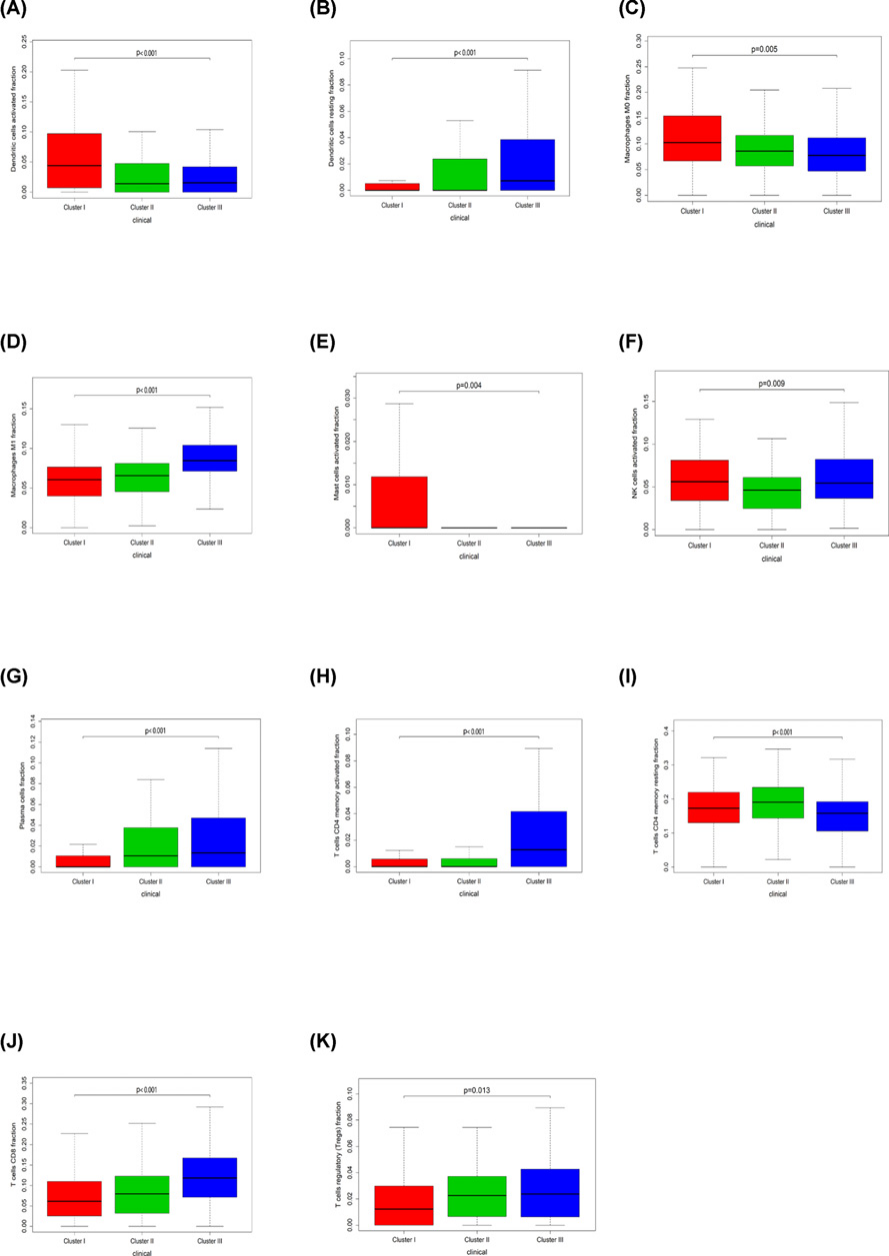
Box plot describing the relationship between immune cell subset and three ovarian cancer clusters (**A–K**) is for Dendritic cells activated, Dendritic cells resting, Macrophages M0, Macrophages M1, Mast cells activated, NK cells activated, Plasma cells, T cells CD4 memory activated, T cells CD4 memory resting, T cells CD8, and T cells regulatory (Tregs), respectively.

The molecular subtypes of OvCa exhibit differences in Clusters I, II, and III subtypes, indicating the activation of specific signaling pathways and different prognosis. Kruskal–Wallis test was used to identify LM21 immune cells in each subtype. In terms of subtype-specific immune cells in OvCa, Cluster I had high levels of Dendritic cells activated, Macrophages M0, Mast cells activated. Cluster II was enriched by T cells CD4 memory resting. Cluster III displayed high levels of Dendritic cells resting, Macrophages M1, NK cells activated, Plasma cells, T cells CD4 memory activated, T cells CD8, T cells regulatory (Tregs) (Figure 11).

### Identification of subtype-specific GO and pathway in subtypes

**Figure 12:**
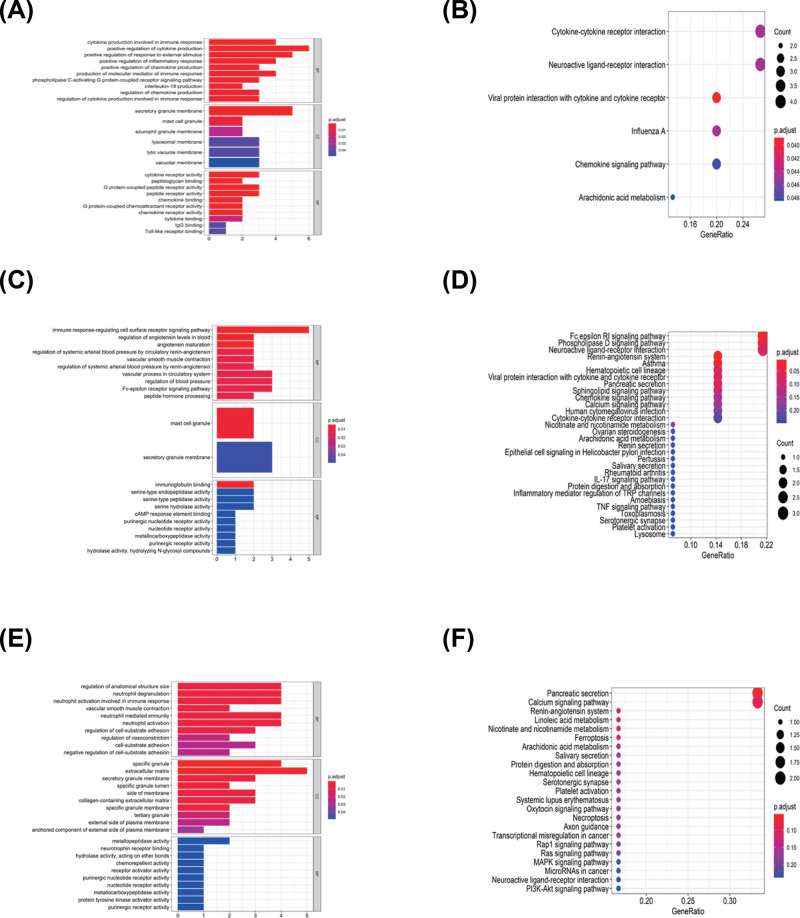
GO and KEGG analysis for three ovarian cancer clusters (**A** and **B**) is for Cluster I, (**C** and **D**) is for Cluster II, and (**E** and **F**) is for Cluster III.

Functional enrichment analysis for DEGs was performed in CI versus CII, III, CII versus CI, III, and C III versus CI, II. For CI versus CII, III, 97 GO terms of biological process, 6 GO terms of cellular component, and 26 GO terms of molecular function were identified with the cutoff point of adjusted *P* value <0.05. Comparison of subgroup II with other groups had identified 32 GO terms of biological process, 2 GO terms of cellular component, and 17 GO terms of molecular function. Comparison of subgroup III with other groups revealed 92 GO terms of biological process, 20 GO terms of cellular component, and 15 GO terms of molecular function. The top GO terms included cytokine activity, immune/inflammatory response, and chemokine activity (Figure 12A,C,E).

All pathways generated by KEGG analysis are related to immune response (Figure 12B,D,F). Cluster I was associated with the cytokine–cytokine receptor interaction, chemokine signaling pathway, and Viral protein interaction with cytokine and cytokine receptor. Cluster II was enriched for IL-17 signaling pathway, TNF signaling pathway, and cytokine–cytokine receptor interaction, and Cluster III was related to MAPK signaling pathway, PI3K-Akt signaling pathway, and MicroRNAs in cancer.

**Figure 13:**
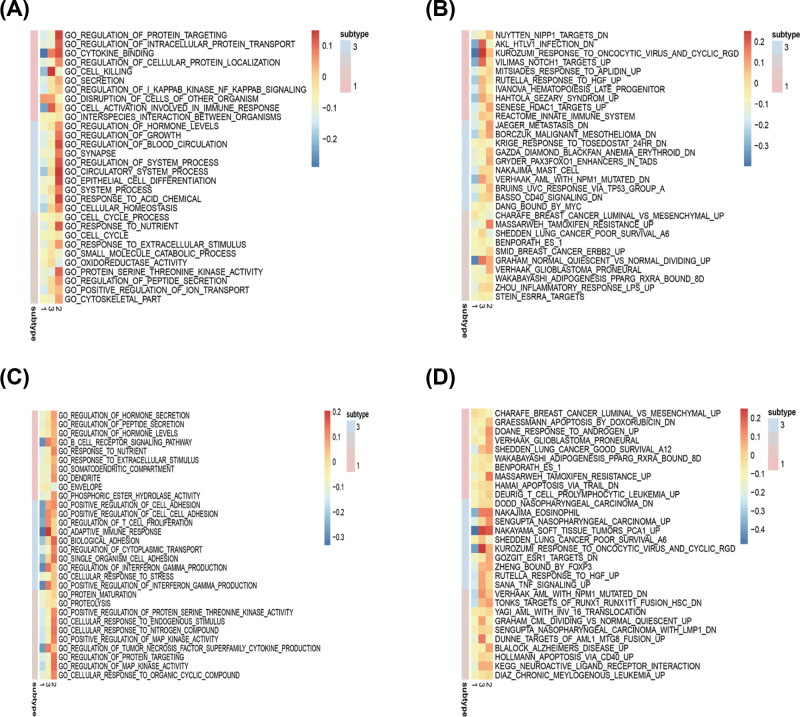
Comparison of functional enriched analysis among three OvCa subgroups (**A**) Up-regulated GO terms; (**B**) Up-regulated KEGG pathways; (**C**) Down-regulated GO terms; (**D**) Down-regulated KEGG pathways.

GSEA analysis was performed to identify gene sets enriched in each subtype. The results revealed distinct enriched gene sets between subtypes. The number of enriched pathways progressively increased from Cluster I through Cluster II. Up-regulated genes for CI–CIII were selected to build a pathway heatmap, which revealed the distinct gene sets enriched in each subtype. Cluster I was associated with ZHENG_FOXP3_TARGETS_IN_THYMUS_UP, NUYTTEN_NIPP1_TARGETS_DN, and CHARAFE_BREAST_CANCER_LUMINAL_VS_MESENCHYMAL_UP; Cluster II was enriched for JAEGER_METASTASIS_DN, NAKAJIMA_MAST_CELL, and BASSO_CD40_SIGNALING_DN; and the KEGG terms in Cluster III were opposite to Cluster I (Figure 13A–D).

### Prediction for immunotherapy and anticancer drug response

**Figure 14:**
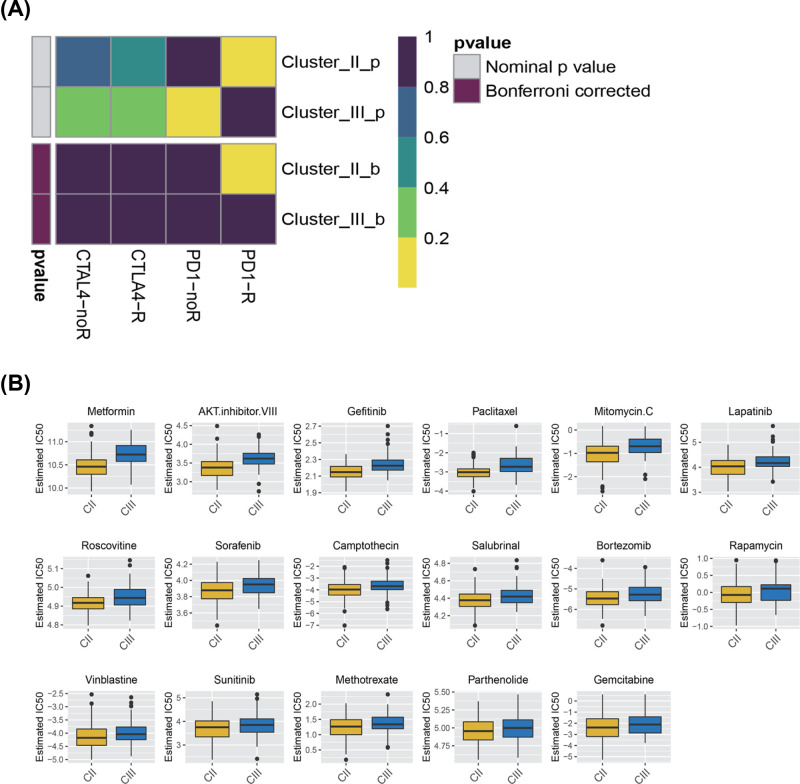
Inferred predictive response for immunotherapy and anti-cancer drugs among three ovarian cancers subtypes (**A**) Submap analysis revealing that Cluster II would be more sensitive to checkpoints PD-1 immunotherapy (Bonferroni-corrected *P*<0.05). (**B**) Box plots manifesting the estimated IC 50 for anti-cancer drugs.

Immunotherapy utilizing monoclonal antibodies against the immune checkpoint inhibitor (ICPC) shows potential application for OvCa. Cluster II responded better to treatment with ICPC compared with cluster III according to TIDE algorithm (*P*<0.05). The expression profiles of OvCa subtypes were compared with another published data set using subclass mapping for specific immune checkpoint. The results suggested that the patients in Cluster II were highly sensitive to programmed cell death 1 (PD1) immunotherapy (Bonferroni correction *P* <0.05) (Figure 14A).

Antitumor drug therapy is one of the basic treatments for patients with OvCa. Prediction model was employed to assess the response to anticancer drugs between two molecular subgroups based on the GDSC cell line dataset. The prediction accuracy of the approach was confirmed with 10-fold cross-validation, and the response sensitivity was estimated with IC50. Among the 130 types of anticancer drug response, 17 exhibited difference between the two clusters. The result suggested that Cluster II was more sensitive to these 17 kinds of drugs than Cluster III. Metformin and AKT inhibitor VIII, paclitaxel, exhibit great potential treatment value for OvCa (*P*<0.05) (Figure 14B). In particular, paclitaxel as the first-line chemotherapy has been widely used in clinical practice.

## Discussion

OvCa has the worst prognosis among gynecological tumors. Hence, it is necessary to find new therapeutic targets to improve the prognosis of OvCa, and make more efforts in this field to reveal the role of immune infiltration in OvCa. In general, immune cells recognize and attack cancer cells to suppress their growth. However, cancer cells have an immune escape mechanism that complicates the genesis and development of tumors. Mounting evidence suggests that tumor immune infiltration is associated with OvCa prognosis [20–22]. Tumor immune microenvironment is a complicated immune system, and further understanding the mechanisms of TIICs in tumor may lead to the discovery of prognostic biomarkers and new targets for tumor treatment.

The present study evaluated the difference of immune cell infiltration in normal and OvCa tissues by utilizing the CIBERSOFT method. Considerable differences in immune cell fraction were found between tumor and normal samples. TIIC can interact with tumor cells to form TME and affect clinical outcomes. Our work revealed that the interaction among TIICs was weakened in the tumor samples than in the normal samples. This finding provides further evidence for the escape of immune surveillance. Moreover, B cells memory, T cells CD4 memory resting, T cells CD4 memory activated, T cells gamma delta, T cells regulatory (Tregs), Macrophages M0, Macrophages M1, Dendritic cells activated and Dendritic cells resting were mainly enriched in tumor tissues. By contrast, B cells naive, plasma cells, T cells CD8, T cells follicular helper, NK cells resting, Monocytes, Macrophages M2, and Mast cells resting were increased in the normal tissues.

Previous studies have shown that immune cell infiltration plays an important role in the prognosis of various solid tumors [7,9,23,24]. In univariate Cox analysis, T cells CD4 memory activated and Macrophages M1 were associated with improved OS. Tumor-related macrophages were the most abundant immune stromal cells in the tumor microenvironment and an important regulator of tumor progression [25]. Macrophages exhibit great plasticity in their phenotype and function and can be polarized into two mainstreams, namely, classically activated macrophage (M1) and alternatively activated macrophage (M2) [26]. M1 macrophages could exert a pro-inflammatory function, boost the Th1 response, and identify and arrest tumor cells; therefore, they emerged as the most prognostic immune cell type in OvCa [27–29]. The subsets of memory T cells include CD4^+^ and CD8^+^ memory T cells. CD8^+^ memory T cells can eliminate tumor cells through the secondary recognition of tumor-associated antigen. CD4^+^ memory T cells inhibit the growth of tumor cells by promoting the proliferation of CD8^+^ memory T cells [30,31]. Increasing studies suggest that T cells CD4 memory activated are associated with improved prognosis, such as in pancreatic adenocarcinomas, breast cancer, cervical cancer, and non-small cell lung cancer [24,32–34]. Our present study further revealed that T cells CD4 memory activated were correlated with increased OS and play vital roles in anti-tumor process. In addition, T cells gamma delta were associated with improved OS according to the Kaplan–Meier curve plot. γδ T cells have received attention because of their potential tumor cytotoxicity [35]. In this work, resting DCs and resting memory CD4 T cells were associated with the degree of malignancy in OvCa. With malignancy progression, the expression of resting DCs decreased gradually. DCs are recognized as the most potent antigen-presenting cells that stimulate naive resting cells and initiate primary immune response. Therefore, we hypothesize that adjusting the immune microenvironment can produce a therapeutic effect in patients with OvCa.

Unsupervised hierarchical clustering analysis utilizing SNFCC+ method uncovered the three specific immunologic subtypes of OvCa and their strong correlation with clinical outcome. Cluster III had better prognosis compared with Clusters I and II. Moreover, each cluster possessed its own distinct functional enrichment terms. The difference of molecular characteristics in three subtypes would alter the immune infiltration patterns. These findings revealed that patients classified under these three molecular subgroups have diverse sensitivity to immune checkpoints. TIDE algorithm indicated that patients in Cluster II had better response to anti-PD1 immunotherapy than those in Cluster III. This discovery provides a possible target for tailored patient treatment, although the specific immune-evasion mechanisms are still unclear. We speculate that Cluster II may be more sensitive to anti-cancer drugs than Cluster III based on the GDSC dataset. The above discussion implicates that the heterogeneity of tumor immune microenvironment would generate difference responses to immunotherapy or anti-cancer drugs. As our study results were derived from bioinformatic analysis, further clinical studies are needed to confirm these results.

In conclusion, the LM22 immune cell profiling based on the CIBERSORT algorithm characterized the landscape of OvCa immune cells. A vital prognostic value associated with clinical outcomes was also revealed. The pattern of LM22 immune cell infiltration in TME of different subtypes initiate the possibility to identify patients who would be sensible to respond to immunotherapy and point out the potential new anti-cancer drug targets for ovarian cancer.

## Data Availability

Data availability could be obtained from TCGA website.

## Abbreviations

OvCa: ovarian cancer
TCGA: The Cancer Genome Atlas
TIIC: tumor-infiltrating immune cell
Treg: T cells regulatory
